# Lung Tumor Cell-Derived Exosomes Promote M2 Macrophage Polarization

**DOI:** 10.3390/cells9051303

**Published:** 2020-05-24

**Authors:** Alexandra Pritchard, Sultan Tousif, Yong Wang, Kenneth Hough, Saad Khan, John Strenkowski, Balu K. Chacko, Victor M. Darley-Usmar, Jessy S. Deshane

**Affiliations:** 1 Department of Medicine, Division of Pulmonary Allergy and Critical Care Medicine, University of Alabama at Birmingham, Birmingham AL 35294, USA; alexandrapritch@gmail.com (A.P.); sana80@uab.edu (S.T.); yongwang@uabmc.edu (Y.W.); kenneth@egtech.us (K.H.); saadk@uab.edu (S.K.); jgstren@uab.edu (J.S.); 2 Mitochondrial Medicine Laboratory, Department of Pathology, University of Alabama at Birmingham, Birmingham AL 35294, USA; baluchacko@uabmc.edu (B.K.C.); vdarleyusmar@uabmc.edu (V.M.D.-U.)

**Keywords:** exosomes, macrophage polarization, M2 macrophages, myeloid-derived suppressor cells, tumor associated macrophages, lung cancer

## Abstract

Cellular cross-talk within the tumor microenvironment (TME) by exosomes is known to promote tumor progression. Tumor promoting macrophages with an M2 phenotype are suppressors of anti-tumor immunity. However, the impact of tumor-derived exosomes in modulating macrophage polarization in the lung TME is largely unknown. Herein, we investigated if lung tumor-derived exosomes alter transcriptional and bioenergetic signatures of M0 macrophages and polarize them to an M2 phenotype. The concentration of exosomes produced by p53 null H358 lung tumor cells was significantly reduced compared to A549 (p53 wild-type) lung tumor cells, consistent with p53-mediated regulation of exosome production. In co-culture studies, M0 macrophages internalized tumor-derived exosomes, and differentiated into M2 phenotype. Importantly, we demonstrate that tumor-derived exosomes enhance the oxygen consumption rate of macrophages, altering their bioenergetic state consistent with that of M2 macrophages. In vitro co-cultures of M0 macrophages with H358 exosomes demonstrated that exosome-induced M2 polarization may be p53 independent. Murine bone marrow cells and bone marrow-derived myeloid-derived suppressor cells (MDSCs) co-cultured with lewis lung carcinoma (LLC)-derived exosomes differentiated to M2 macrophages. Collectively, these studies provide evidence for a novel role for lung tumor-exosomes in M2 macrophage polarization, which then offers new therapeutic targets for immunotherapy of lung cancer.

## 1. Introduction

Lung cancer continues to be the leading cause of cancer-associated morbidity and mortality among men and women, claiming ~1.3 million deaths annually worldwide [1,2,3]. The predominant form of lung cancer is non-small-cell lung carcinoma (NSCLC), which accounts for about 85 percent of all lung cancers. The lung tumor microenvironment is complex and is composed of both tumor-promoting and tumor-inhibiting cells. Tumors at their developmental stage actively drive the generation of regulatory and immunosuppressive immune cells such as tumor associated macrophages (TAMs), myeloid-derived suppressor cells (MDSCs), and regulatory lymphocytes that promote tumor growth by suppressing anti-tumor immunity [4,5,6,7]. During cancer progression, abundant infiltration of macrophages occurs in the TME [8,9] and is associated with poor prognosis for cancer patients [10]. Macrophages adopt diverse phenotypes and differentiate into different subsets, including M1 (classically activated macrophages) and M2 (alternatively activated macrophages) [11] in response to various stimuli. M1 macrophages act against infectious microbes and induce Th1 (Type 1 helper T cells) through the production of pro-inflammatory cytokines [11,12]. In contrast, the M2 macrophages inhibit the Th1 immune response, promote Th2 (Type 2 helper T cells) immunity, and participate in wound healing and tissue remodeling [11,13].

TAMs accelerate tumor growth by promoting tumor angiogenesis, metastasis, and also by modulating T cell activation and differentiation [14,15,16]. TAMs exhibit characteristics of M2 macrophages, which participate in tumor progression by suppressing anti-tumor immunity [17]. However, the molecular mechanisms underlying their differentiation and/or infiltration in TME have not been investigated in detail. Classically activated or M1 macrophages and alternatively activated or M2 macrophages also maintain distinct metabolic profiles. M1 mainly obtain their energy by glycolysis while M2 macrophages are known to be more dependent on aerobic metabolism [18,19]. The difference in metabolic dependency regulates the induction of M1 and M2 polarization. Artificial enhancement of oxidative metabolism in macrophages induced M2 polarization, whereas inhibition promoted M1 polarity [18,19]. The metabolic signatures in the TME thus favors the differentiation and survival of TAMs.

Cellular cross-talk in the TME is a central driver of tumor progression. Extracellular vesicles called exosomes have emerged as significant players in the immune-stromal cross-talk in the TME. Exosomes are small 50–200 nm membraned vesicles released by a variety of cell types in the extracellular milieu [20,21,22,23]. Exosomes are involved in both local and systemic cell-cell communication by transfer of active biomolecules (nucleic acid, lipids, and proteins) and, as we have recently shown, mitochondria [24,25,26,27,28]. Tumor cell-derived exosomes are emerging as regulators of tumor growth [29], tumorigenesis [25], tumor invasion, and metastasis [25,30]. Tumor cell-derived exosomes have been reported to activate or suppress immune cells by activating molecular signals that directly or indirectly influence anti-tumor immune responses [31,32]. It is well known that tumor cell exosomes are taken up by bone marrow-derived macrophages (BMDMs). Tumor cell-derived exosomes regulate the differentiation of myeloid progenitor cells [33], and also are known to alter macrophage phenotype by transferring the IL-6 receptor gp130 [34]. However, the functional impact of these tumor exosomes on macrophage polarization is not clear. There is emerging evidence that cancer cell-derived exosomes direct metabolic reprogramming of the host’s immune cells [35]. Therefore, tumor-derived exosomes may play a decisive role in the reprogramming of macrophage metabolism in TME and redirect their polarization. In support of this hypothesis, we show that lung tumor cell-derived exosomes are internalized by non-committed macrophages (M0) in a time-dependent manner, which are then polarized to the M2 macrophage phenotype. Using a p53 null human lung cancer cell line (H358), we demonstrate that p53 modulates exosome production by human lung tumor cells, and the exosome-mediated M2 polarization is p53 independent. Importantly, we show that cancer-derived exosomes modulate transcriptional changes and reprogram macrophage metabolism and promote their differentiation to an M2 phenotype. Additionally, our studies show that tumor cell-derived exosomes promote differentiation of immunosuppressive MDSCs to M2 macrophages. Collectively, our present study reveals a novel regulatory role for lung cancer-derived exosomes in the lung TME.

## 2. Materials and Methods

### 2.1. Cell Culture

Adenocarcinoma human alveolar basal epithelial cells (A549, ATCCR CCL-85TM) were purchased from the American Type Culture Collection (Manassas, VA, USA) and cultured in DMEM/F12 50/50 supplemented with 10% FBS, 2 mM L-glutamine, 100 IU/mL Penicillin and 100 µg/mL Streptomycin (Corning, Corning, NY, USA), until 100% confluency. H358 (p53 null human lung cancer) cells (ATCCR CCL-5807TM) were cultured in 4.53 gm/L dextrose containing DMEM supplemented with 10% FBS, 1% L-glutamine, and 1% penicillin/streptomycin (P/S), non-tumor epithelial cells HEK293 (ATCCR CCL-1573TM) and syngeneic lewis lung carcinoma (LLC) cells (ATCCR CCL-1642TM) were cultured in DMEM supplemented with 10% FBS, 1% L-glutamine, and 1% penicillin/streptomycin (P/S). Then the culture media were replaced with exosome-depleted media in preparation for exosome isolation. THP-1 cells (ATCCR TIB-202TM) were cultured in RPMI-1640 media (Gibco, Waltham, MA, USA) modified with 10% FBS, 1% L-glutamine, 0.002M sodium pyruvate, 2M HEPES, penicillin/streptomycin (P/S) and 4.5 g/L of dextrose,. THP-1 cells were then stimulated with 20–100 ng/mL of phorbol 12-myristate 13-acetate (PMA) overnight to transform them into non-committed M0 cells. M0 cells were then used to study tumor cell-derived exosome-mediated M2 polarization and bioenergetics studies. All the cell lines were first tested for mycoplasma contamination by following the manufacturer’s protocol of a PlasmoTest™ - Mycoplasma Detection Kit (cat.code: rep-pt1 Invivo-Gen, San Diego, CA, USA). Only mycoplasma negative cells were used for the experiments.

### 2.2. M1 and M2 Polarization

For M1 polarization, THP-1 cells were stimulated with 25 ng/mL PMA for 24 h, and then the cells were further stimulated with LPS (100 ng/mL, Sigma-Aldrich, St. Louis, MO, USA) and IFN-γ (20 ng/mL, PeproTech, Rocky Hill, NJ, USA) for 72 h. For M2 polarization, THP-1 cells were stimulated with 25 ng/mL PMA for 48 h, followed by resting in RPMI-1640 medium without PMA for 48 h. Then the cells were further stimulated with IL-4 (50 ng/mL, PeproTech) and IL-13 (20 ng/ML, PeproTech) for 48 h [36,37].

### 2.3. Isolation of Exosomes

Exosomes were isolated from conditioned media of cultured A549 cells. Exosome-depleted A549 cell culture media (160 mL) were centrifuged at 300× *g* for 10 min at 4 °C to separate the supernatant from debris. The supernatant was collected and centrifuged at 2000× *g* for 10 min at 4 °C to separate the supernatant and any apoptotic bodies. The supernatant was collected again and centrifuged for 30 min at 10,000× *g* at 4 °C in ultra-centrifugation tubes in a 70.0 Ti rotor. After centrifugation, the supernatant was filtered through a 0.2 μm cellulose acetate filter (Corning). The filtered supernatant was ultra-centrifuged again at 100,000× *g* for 70 min at 4 °C. The re-suspended pellet was washed with PBS at 100,000× *g* for 70 min at 4 °C. The pellet produced from the wash was re-suspended in 100 µL of PBS and stored at −80 °C.

### 2.4. NanoSight Analyses of Exosomes

The mean concentration and mean size of the exosomes were measured using a NanoSight NS300 (Malvern Panalytical, Westborough, MA, USA)). Before running the sample, the machine was calibrated with 100 nm polystyrene latex microspheres (Malvern Instruments Ltd., Malvern, UK). One mL of exosomes diluted 100-fold with PBS was collected in a 1 mL syringe. The syringe was inserted on the syringe pump of the Nanosight. The exosomes were injected at a flow rate of 25 at room temperature. 5 videos were acquired for each sample. All videos were acquired at a temperature of 23.2–23.3 °C, viscosity: 0.923–0.927 cP; camera level: 7; capture duration: 1 min/video; shutter speed of 11.12 ms; camera type: SCMOS; gain: 1; minimum tracks completed: 2000–4000/video; frames processed: 1951/video; frames per second: 32.5 fps; blur: auto; and detection threshold: 5.

### 2.5. Labeling Exosomes with PKH26

Exosomes (1 × 10^7^) were stained with the lipophilic dye PKH-26 (Sigma-Aldrich, St. Louis, MO, USA) following the manufacturer’s recommendations. Briefly, 1 mL diluent C was mixed with 1 μL of PKH-26, and the exosomes diluted in 1 mL diluent C were added. The exosomes and stain solution were incubated at 37 °C for 4 min in the dark. The labeling reaction was stopped by adding an equal volume of FBS. Next, 0.5 volume of Invitrogen exosome isolation media were added. The mixture was vortexed and incubated overnight at 4 °C in the dark. The exosomes were washed the following day at 10,000× *g* for 60 min. The pellet was then re-suspended in PBS.

### 2.6. Co-Culture of Exosomes with THP-1 Cells

Exosomes were co-cultured with M0 macrophages at a ratio of 10 exosomes per cell in 12-well plates in a 1 mL per well total volume (3 replicate wells) for periods of 24 h, 48 h, or 72 h. After the co-culture period, the macrophages were collected and processed for ImageStream and flow cytometry analysis. For bioenergetics experiments, the exosomes and macrophages were co-cultured in a 10:1 ratio in 96-well plates with 100 μL per well and 5–6 replicates per condition.

### 2.7. Flow Cytometry

After macrophages were co-cultured with exosomes for 24 h, 48 h, or 72 h, macrophages co-cultured with stained exosomes were then stained with CD64 Percp-cy5 (clone 10.1, BD Pharmingen, San Jose, CA, USA), CD206 Alexa Flour 488 (clone 19.2, eBioscience, Waltham, MA, USA), CD163 Pecy7 (clone eBioGHI/61, Thermo Fisher Scientific, Waltham, MA, USA) and CD11b APCcy7 (clone M1/70, BD Bioscience, San Jose, CA, USA). After staining, the cells were washed twice with PBS. Flow cytometry data were acquired using a BD LSRII (Franklin Lakes, NJ, USA) and data analysis performed with FlowJo X.

### 2.8. ImageStream Flow Cytometry

Macrophages were stained with CD64 Percp-cy5 (clone 10.1, BD Pharmingen), CD206 Alexa Flour 488 (clone 19.2, eBioscience), and CD163 Pecy7 (clone eBioGHI/61, Thermo Fisher Scientific) for ImageStream analyses. For Image Stream, samples were imaged at 60× magnification while acquiring data on different channels. The machine was calibrated using single color stained and unstained controls, as well as calibration beads to adjust the noise levels and spectral compensation. Brightfield data were collected on channels Ch01 and Ch09, and side-scatter was collected on Ch12. 5000 events were acquired and used for analysis in IDEAS software version 6.2 (EMD Millipore, Billerica, MA, USA).

### 2.9. qRT-PCR

RNA was isolated using Pure LinkTM RNA Mini Kit (cat #12183018A0, Invitrogen, Carlsbad, CA, USA). cDNA was synthesized from RNA by using the Prime Script 1st Strand cDNA Synthesis Kit (Takara Bio, Shiga, Japan). Forward and reverse primers for IL-10, Fizz 1, Ym1 and Arg-1 were combined with VeriQuest SYBR Green qPCR Master Mix (Affymetrix, Santa Clara, CA, USA) and H2O in a 96 well plate to perform Real-Time PCR by using the StepOnePlus™ Real-Time PCR System (cat # 4376600, Applied Biosystems, Foster City, CA, USA). The housekeeping gene was Ribosomal protein S6 (RPS 6). Fold change was calculated by using the Livak-Schmittgen method (Delta Delta CT method) [38]. The following primers were used to assess the M2 gene structure (Table 1).

### 2.10. Bioenergetics Assay

M0 macrophages were cultured at a density of 20,000 cells/well in Xf-96-well plates for cellular bioenergetics measurement using the Seahorse Extracellular Flux Analyzer (Agilent, Santa Clara, CA, USA) which simultaneously measures oxygen consumption rates and extracellular acidification in live cells. Following complete differentiation of THP-1 cells, the M0 macrophages were co-cultured with or without exosomes for 24 h, washed using XF-DMEM assay media, and allowed to equilibrate in a non-CO_2_ incubator for 1 h at 37 °C prior to assay. The mitochondrial stress test was performed by sequentially injecting oligomycin (Oligo, 1 µg/mL), carbonyl cyanide 4-(trifluoromethoxy)-phenylhydrazone (FCCP, 0.3 µM), antimycin A (Anti A, 10 µM) and 2-deoxyglucose (50 mM) [39]. Rotenone, antimycin A or azide were used to determine the oxygen consumption rates (OCR) related to complex I, complex II–III or complex I–IV, respectively [40,41].

### 2.11. Statistical Analyses

Statistical analyses were performed using GraphPad Prism 5 software (La Jolla, CA, USA), and values were presented as mean ± SD. Significant differences between the group means were determined by one-way ANOVA with Tukey’s multiple comparisons test. Comparisons between two groups were evaluated by an unpaired parametric student’s *t*-test. A value of *p* < 0.05 was considered statistically significant.

## 3. Results

### 3.1. Secretion of EpCAM+ Lung Tumor Cell-Derived Exosomes is p53 Dependent

**Figure 1:**
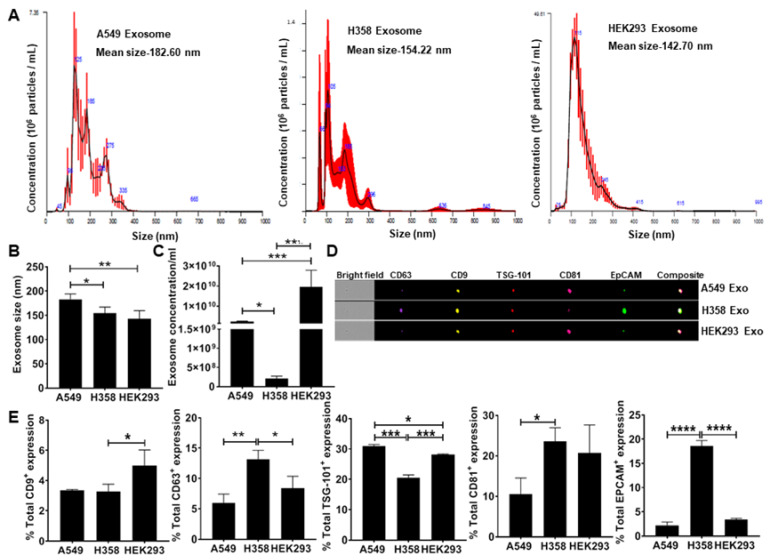
Quantitation and characterization of tumor-cell derived exosomes. NanoSight and ImageStream analyses of A549-derived exosomes determined size, concentration, and characterization. Human lung cancer cells A549, H358, and non-tumor cells HEK293 were cultured in exosome depleted media for 24 h. Exosomes were isolated from cultured supernatant by using differential centrifugation (**A**) Mean size and concentration of A549 cell-derived exosome determined by NanoSight analysis, mean size was recorded as 182.6 nm, 154.22 nm, and 142.7 nm for A549, H358 and HEK293 cells respectively. (**B**) NanoSight data analysis showing the small size of H358 and HEK293 exosomes compared to A549 cell-derived exosomes. (**C**) NanoSight data analysis showing significantly reduced concentration of exosomes produced from 160 mL of culture media collected of p53 null human lung cancer cells H358 compared to A549 and HEK293 cell exosomes. (**D**) Representative Figure of ImageStream analysis to characterize A549 derived exosomes by expression signals of CD63, CD9, TSG-101, CD81, and EpCAM. (**E**) Percentage of total expression of conventional exosomes markers expressed over A549, H358 and HEK293 cell-exosomes respectively * *p* < 0.05, ** *p* < 0.01, *** *p* < 0.001, **** *p* < 0.0001.

To test the hypothesis that lung tumor cell-derived exosomes re-direct macrophage polarization, we first isolated exosomes from conditioned media obtained from adenocarcinoma human alveolar basal epithelial cells (A549) and p53 null human lung cancer cells (H358) including the non-tumor epithelial cell line (HEK293) as a negative control. NanoSight analysis of isolated exosomes confirmed the particle size under a range of 50–200 nm (Figure 1A) consistent with previous observations [22]. The H358 exosomes were significantly smaller than those isolated from A549 (Figure 1B). NanoSight analysis also showed a significantly lower concentration of H358 exosomes compared to A549 and HEK293 exosomes isolated from an equal volume of conditioned media (Figure 1C), suggesting that as reported previously, p53 may regulate exosomes secretion from lung tumor cells [42]. Further ImageStream analysis revealed the expression of epithelial cell adhesion molecule (EpCAM) and exosome specific surface markers, including CD9, CD63, tumor susceptibility gene 101 (TSG-101), and CD81 (Figure 1D,E) confirming the tumor cell-derived vesicles as exosomes.

### 3.2. Internalization of Tumor Cell-Derived Exosomes by Macrophages is Time-Dependent

To examine if lung tumor cell-derived exosomes are internalized by macrophages, we first pre-labeled exosomes with the lipophilic PKH red dye (PKH-26), co-cultured them with THP-1 M0 macrophages at a 10:1 ratio (10 exosomes/THP-1-M0) for 24–72 h and then analyzed their internalization by macrophages.

**Figure 2:**
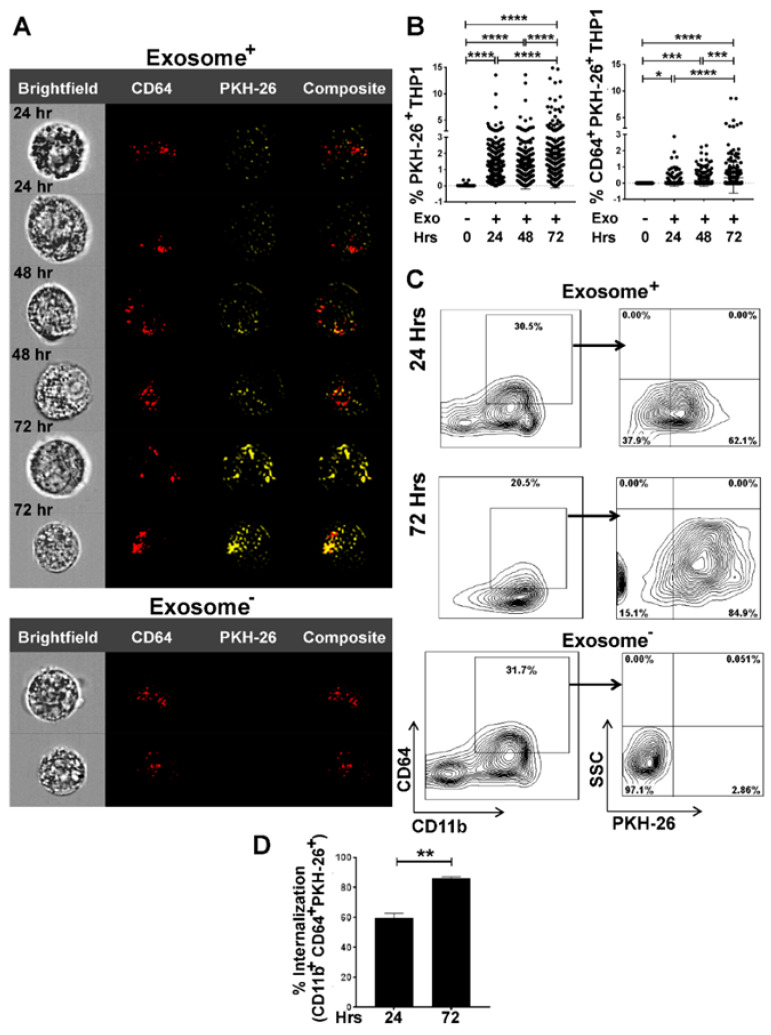
ImageStream and flow cytometry analyses quantify time-dependent internalization of tumor cell-derived exosomes by THP-1 cells. THP-1 cells were seeded and differentiated into M0 macrophages upon overnight stimulation with PMA (20–100 ng/mL). M0 macrophages were then co-cultured with PKH-26 stained A549-derived exosomes in 10:1 ratio (10 exosomes/cell) for 24 to 72 h. Image Stream analysis showing Bright field, CD64^+^, PKH-26^+^, and composite images. (**A**) Time-dependent internalization of exosomes by CD64^+^ populations assessed by internalization of PKH-26 stained A549-derived exosomes. CD64^+^ population, without internalization indicates M0 phenotype. (**B**) MATLAB analysis of percentage of PKH-26^+^ and CD64^+^PKH-26^+^ signals to show time-dependent internalization of exosomes. Fluorescent signals were collected from 300 cells for each time point. (**C**) Representative flow cytometry of exosome internalization analysis at 24 h and 72 h. Group comparisons of 24 h exosome-, 24 h exosome+, and 72h exosome+ were made. After co-culture, cells were prepared for flow cytometry and stained with CD64, CD11b, and PKH-26. The experiment was repeated twice, with three replicates per sample in each experiment. Exosome- sample was used as control (**D**) Percentage of internalization by THP-1 macrophages as CD11b^+^CD64^+^PKH-26^+^ showing a significant increase of uptake in 72 h compared to 24 h * *p* < 0.05, ** *p* < 0.01, *** *p* < 0.001, **** *p* < 0.0001.

As indicated by the PKH-26 signal in exosomes+ samples compared to exosomes- analyzed by ImageStream analysis, exosomes were internalized by macrophages in a time-dependent manner (Figure 2A). Furthermore, we estimated the intensity of the PKH-26 red dye signals for exosome internalization by MATLAB analysis, which confirmed a time-dependent uptake of exosomes (Figure 2B).

Dot plots from ImageStream flow cytometry analyses were consistent with the MATLAB data also supporting a time-dependent exosome internalization (Supplementary Figure S1A,B). Flow cytometry analyses confirmed the internalization of PKH red labeled exosomes by THP-1-M0 macrophages, as evidenced by increased expression of PKH-26 in CD64^+^ population (Figure 2C,D).

### 3.3. Lung Tumor Cell-Derived Exosomes Promote M2 Macrophage Polarization in a Time-Dependent Manner

Since tumor cell-derived, exosomes have been shown to regulate macrophage phenotype resulting in a pro-tumorigenic TME [34], we next evaluated the impact of lung cancer-derived exosomes on macrophages. We isolated exosomes from the conditioned media of A549, and cultured PMA-stimulated THP-1-M0 macrophages in the presence or absence of freshly isolated exosomes in 10:1 (10 exosomes/cell) ratio for 72 h. 

**Figure 3:**
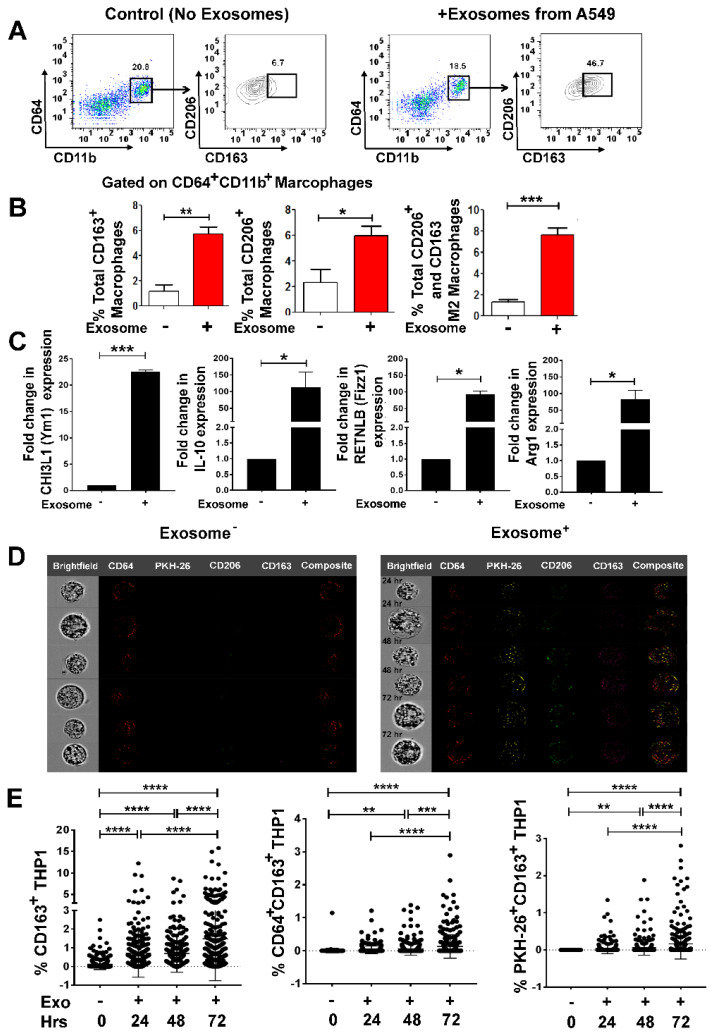
A549 cell-derived exosomes differentiate non-committed M0 macrophages to M2 phenotype. A549 human lung cancer cells were cultured in exosome depleted media for 24 h. Exosomes were isolated from cultured supernatant using differential centrifugation. Exosomes were stained with PKH-26 for overnight. THP-1 cells were seeded and transformed into M0 macrophages upon overnight stimulation with PMA (20–100 ng/mL). M0 macrophages were then co-cultured with PKH-26 stained, A549 derived exosomes in 10:1 ratio (10 exosomes/cell) for 24 to 72 h. (**A**) Representative flow cytometry plot showing in-vitro induction of M2 phenotype (CD11b^+^CD64^+^CD163^+^CD206^+^) with A549-derived exosomes. (**B**) Left to right panel-Total percentage of CD163^+^ macrophages, showing significant increase in CD163^+^ macrophages that have internalized PKH^+^ exosomes. Total percentage of CD206^+^ macrophages, showing significant induction in CD206^+^ macrophages that have internalized PKH^+^ exosomes. Total percentage of M2 macrophages as CD11b^+^CD64^+^CD163^+^CD206^+^ showing significantly induced M2 phenotypes with exosome-positive sample. (**C**) THP-1 cells were seeded and transformed into M0 macrophages upon overnight stimulation with PMA (20 ng/mL). M0 macrophages were then co-cultured with A549 derived exosomes in 10:1 ratio (10 exosomes/cell) for 24 h M2 gene signature, CHI3L1 (Ym1), IL-10, RETNLB (Fizz1), and Arg1 were upregulated on 24 h of A549-derived exosomes treatment, assessed by qRT-PCR in the cells. (**D**) ImageStream analyses showing CD64^+^, PKH-26^+^, CD206^+^ CD163^+^ macrophages before and after internalization of PKH+ exosomes. Left panel shows composite images from several M0 macrophages without exosome internalization. The right panel shows composite images of M0 macrophages with exosome internalization that have polarized to become M2 (CD206^+^ CD163^+^) (**E**) Time-dependent increase of M2 polarization induced by A549-derived exosomes showing by percentage of CD163^+^, CD64^+^CD163^+^ and PKH-26^+^CD163^+^ analyzed by using MATLAB analysis with the signals collected from 300 cells in each time points. * *p* < 0.05, ** *p* < 0.01, *** *p* < 0.001, **** *p* < 0.0001.

We characterized exosome-mediated M2 polarization by flow cytometry using M2 specific markers (CD163 and CD206) as reported previously [43,44,45]. A549 exosomes enhanced M2 polarization in M0 macrophages as evidenced by significantly higher expression of CD163^+^, CD206^+^ and CD163^+^CD206^+^ in the CD11b^+^CD64^+^ gated population (Figure 3A,B). To ascertain whether M2 polarization is lung tumor exosome specific, RT-qPCR was performed to examine the M2 gene signature [44,45].

PMA stimulated THP-1-M0 macrophages are presumed as controls or reference that have not received signals for M2 polarization. M2 macrophage polarization was assessed by measuring the expression of several M2 specific gene signatures: chitinase-3-like protein 1 (CHI3L1) (Ym1), interleukin-10 (IL-10), resistin-like beta (RETNLB) (Fizz1), and arginase-1 (Arg-1), which were significantly upregulated on exosome addition to M0 macrophages (Figure 3C). 

To further confirm if M2 polarization was indeed due to internalization of lung tumor exosomes by M0 macrophages, we pre-labeled exosomes with PKH-26 red dye and co-cultured them with THP-1-M0 macrophages in 10:1 (10 exosomes/THP-1-M0) ratio for 24–72 h and analyzed for M2 polarization. Notably, a time-dependent increase in the signals for M2 markers CD163 and CD206 was observed with the internalization of red PKH-26 stained exosomes (Figure 3D, Figure S1C). Furthermore, we compared the intensity of CD163^+^, CD64^+^CD163^+^, PKH-26^+^CD163^+^ in exosomes-positive samples by MATLAB analysis, which also showed a time-dependent M2 polarization with exosomes internalization (Figure 3E). Collectively these data show that M0 macrophages internalize lung tumor exosomes and polarize the cells to an M2 phenotype.

### 3.4. Non-Tumor Cell-Derived Exosomes are Unable to Polarize M0 to M2 While M2 Macrophage Polarization by Lung Tumor Cell-Derived Exosomes May Not Be p53 Dependent

**Figure 4:**
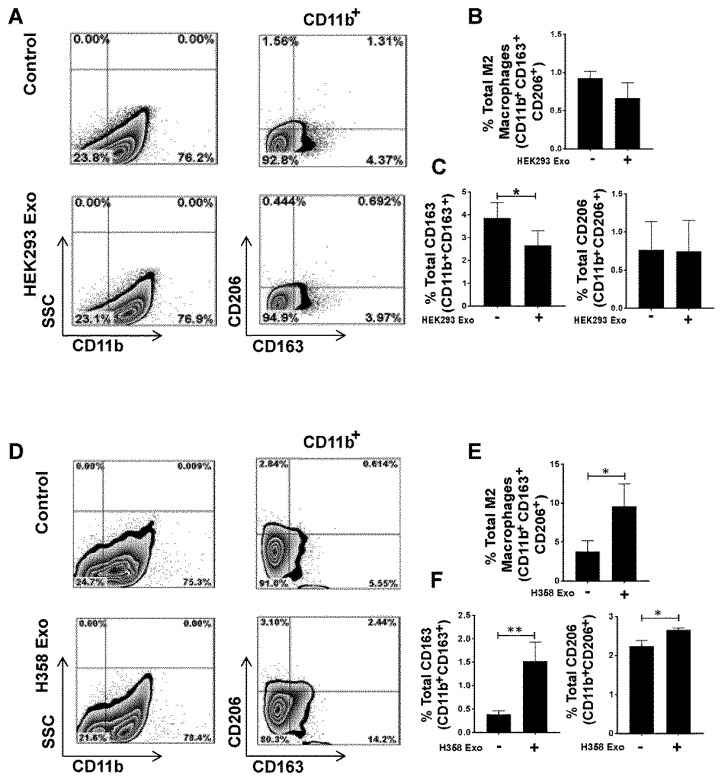
Non-tumor cell-derived exosomes are unable to promote M2 polarization but tumor cell-derived exosome-mediated M2 polarization is p53 independent. THP-1 cells were seeded and differentiated into M0 macrophages upon overnight stimulation with PMA (20–100 ng/mL). M0 macrophages were then co-cultured with non-tumor cell HEK293 and p53 null H358-derived exosomes in 10:1 (10 exosomes/cell) ratio for 72 h. (**A**) Representative flow cytometry plot showing lack of in vitro induction of M2 phenotype (CD11b^+^CD163^+^CD206^+^) with HEK293-derived exosomes. (**B**) Total percentage of M2 (CD11b^+^CD163^+^CD206^+^) macrophages, showing no significant difference with exosome-positive samples. (**C**) Total percentage of CD163+ and CD206+ macrophages, with or without HEK293 exosome-positive samples. (**D**) Representative flow cytometry plot showing in vitro induction of M2 phenotypes (CD11b^+^CD163^+^CD206^+^) with p53 null H358 cell-derived exosomes. (**E**) Total percentage of M2 (CD11b^+^CD163^+^CD206^+^) macrophages, showing a significant difference with H358 cells with exosomes. (**F**) Total percentage of CD163^+^ and CD206^+^ macrophages, with or without H358 exosome-positive samples. * *p* < 0.05, ** *p* < 0.01, *** *p* < 0.001, **** *p* < 0.0001.

The effect of non-tumor exosomes on M0 macrophages was assessed by co-culturing THP-1-M0 macrophages with HEK293 cell-derived exosomes in a 10:1 ratio for 72 h. Flow cytometry analysis, performed with staining of CD11b, CD163, and CD206 antibodies, showed that non-tumor cell-derived exosomes were unable to promote M2 polarization (Figure 4A,B). To further define the link between p53 and the tumor exosome-mediated M2 polarization, THP-1-M0 macrophages were co-cultured with H358 exosomes in a 10:1 (10 exosomes/cell) ratio for 72 h and then stained with CD11b, CD163 and CD206. M2 polarization was induced by H358 exosomes (Figure 4D,E), suggesting that lung tumor cell-derived exosomes induce the M2 phenotype independent of p53. Next, we investigated whether tumor or non-tumor cell exosomes have any impact on M1 polarization. THP-1-M0 macrophages were co-cultured with H358, and HEK293 exosomes in a 10:1 (10 exosomes/cell) ratio and macrophages in the M1 phenotype were also induced as a positive control as reported previously [46]. M1 polarization was assessed by the staining of CD11b, HLA-DR, and STAT-1 antibodies.

As shown in Figure S2A, M1 macrophage polarization was not induced with the tumor or non-tumor exosomes, indicating that lung tumor exosomes polarize M0 macrophages to only M2 phenotype (Figure S2B). Taken together, these results provide evidence that non-tumor cell exosomes are unable to induce M2 phenotype, whereas lung tumor exosomes promote M2 polarization independent of p53.

### 3.5. Murine Lung Tumor Cell-Derived Exosomes Differentiate Bone Marrow (BM) Cells and MDSCs to M2 Macrophages

**Figure 5:**
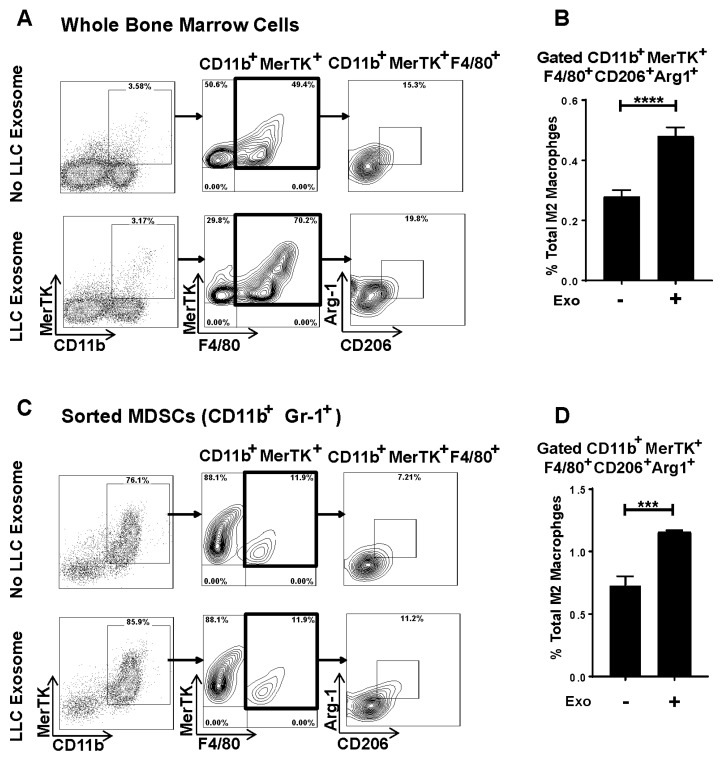
Syngeneic lung cancer cell lewis lung carcinoma (LLC)-derived exosomes induce M2 polarization and also differentiate MDSCs to M2 phenotype. Bone marrow (BM) cells from wild type (WT) mice were seeded then co-cultured with LLC derived exosomes in 10:1 ratio (10 exosomes/cell) for 72 h. Cells were stained with antibodies (CD11b, MERTK, F4/80, Arginase-1 and CD206), and flow cytometry was performed to identify M2 phenotype. (**A**) Representative flow cytometry plot showing in-vitro induction of M2 phenotype (CD11b^+^MERTK^+^F4/80^+^CD206^+^Arginase-1^+^) upon co-culture with LLC cell-derived exosomes. (**B**) Total percentage of M2 macrophages (CD11b^+^MERTK^+^F4/80^+^CD206^+^Arginase-1^+^) showing significantly induced M2 phenotype with LLC exosome+ sample. (**C**) MDSCs (CD11b^+^Gr-1^+^) were immunosorted from BM cells of wild type (WT) mice then co-cultured with LLC-derived exosomes in 10:1 ratio (10 exosomes/cell) for 72 h. Cells were stained with antibodies (CD11b, MERTK, F4/80, Arginase-1 and CD206), and flow cytometry was performed to identify M2. Representative flow cytometry plot showing in-vitro polarization of MDSCs to M2 phenotype (CD11b^+^MERTK^+^F4/80^+^CD206^+^Arginase-1^+^) upon LLC cell-derived exosomes treatment. (**D**) Total percent of M2 macrophages (CD11b^+^MERTK^+^F4/80^+^CD206^+^Arginase-1^+^) showing significantly induced M2 phenotype following internalization of exosomes (*** *p* < 0.001).

To evaluate lung tumor cell-derived exosome-mediated M2 polarization in an immunocompetent syngeneic setting, we first isolated exosomes from conditioned media of syngeneic mouse NSCLC lewis lung carcinoma (LLC). The size and concentration of LLC exosomes were determined by NanoSight analysis (Figure S3). To assess if lung tumor cell-derived exosomes alter the phenotype of BMDMs, LLC exosomes were added to whole bone marrow cells (BMs) at a 10:1 (10 exosomes/cell) ratio for 72 h. LLC exosome-mediated M2 polarization was analyzed by flow cytometry using specific markers (CD206 and Arginase-1) and with staining of CD11b, proto-oncogene tyrosine-protein kinase MER (MerTK), and F4/80 antibodies. LLC exosomes promoted M2 polarization in BMs, evidenced by significantly higher expression of CD206^+^Arg-1^+^ in the CD11b^+^MerTK^+^F4/80^+^ gated population (Figure 5A,B). MDSCs have been shown to be precursors of TAMs and can differentiate to the M2 macrophage phenotype in TME [47,48]. Therefore, we next evaluated if LLC exosomes alter MDSCs’ phenotypes to M2 macrophages. We immune sorted MDSCs as CD11b^+^Gr-1^+^ from BM of WT naïve mice and co-cultured with LLC exosomes at a 10:1 (10 exosomes/cell) ratio for 72 h. To measure M2 polarization, cells were stained with CD11b, MerTK, F4/80, CD206, and Arg-1 and analyzed by flow cytometry. The addition of LLC exosomes promoted the differentiation of M2 macrophages from immune sorted MDSCs (Figure 5C,D), suggesting that lung tumor exosomes change MDSCs to M2 phenotypes. 

### 3.6. Lung Tumor Cell-Derived Exosomes Modulate Cellular Bioenergetics of Macrophages

**Figure 6:**
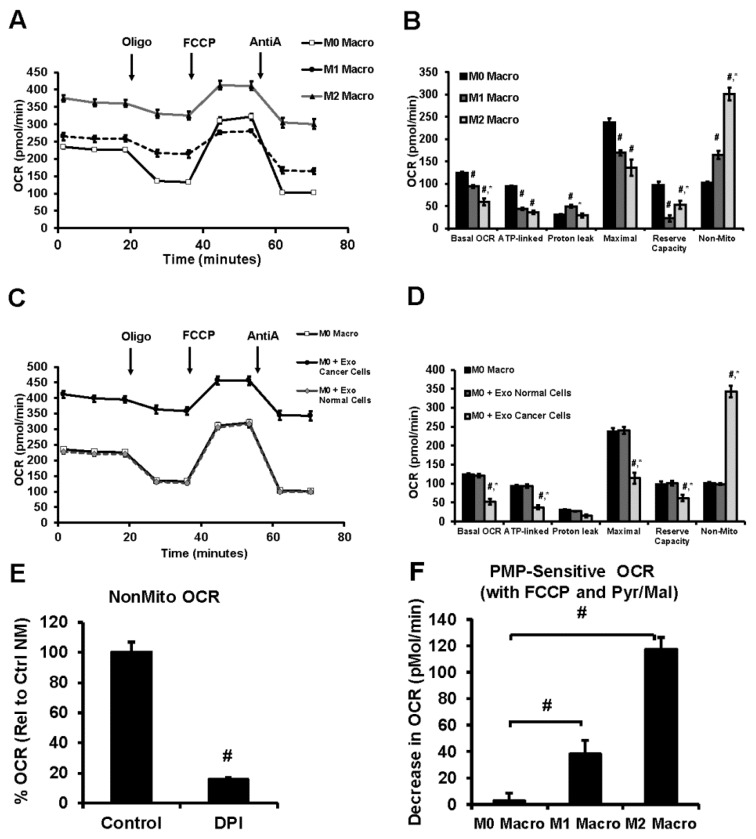
Alterations in cellular bioenergetics of macrophages co-cultured with Tumor-derived exosomes by extracellular flux analysis. (**A**) Cellular bioenergetic profiles and (**B**) the cellular bioenergetic parameters (Basal OCR, ATP-linked OCR, Proton Leak, Maximal OCR, Reserve Capacity and Non-Mitochondrial OCR) of M0, M1 and M2 macrophages as determined by sequential injection of oligomycin (Oligo), FCCP, antimycin A (AntiA). Mean values from 6–8 replicates with ± sem. # *p* < 0.05 relative to M0 macrophages and * *p* < 0.05 relative to M1 macrophages. (**C**) Comparison of the cellular bioenergetic profiles of untreated M0 macrophages and those co-cultured with normal cell (HEK293) or tumor cell (A549)–derived exosomes, and (**D**) quantitation of the bioenergetic parameters of Figure 6C. Mean values from 6–8 replicates with ± sem. # *p* < 0.05 relative to M0 macrophages and * *p* < 0.05 relative to M0-co-culture with normal cells-derived exosomes. (**E**) Inhibition of nonmitochondrial OCR in nonpermeabilized macrophages using DPI. Mean values from 4–8 replicates with ± sem. # *p* < 0.005 compared to M0 macrophages; * *p* < 0.01 compared to M1 macrophages. (**F**) PMP-sensitive mitochondrial OCR in macrophages. Mean values from 3–8 replicates with ± sem. # *p* < 0.05 relative to M0 macrophages and * *p* < 0.05 relative to M0-co-culture with normal cells-derived exosomes.

Modulation of cellular bioenergetic function is considered to be a regulatory mechanism associated with macrophage polarization. As shown in Figure 6A,B, both M1 and M2 macrophages demonstrated a significant decrease in bioenergetic parameters (Basal OCR, ATP-linked OCR, Maximal OCR, and Reserve Capacity) compared to M0 macrophages (Figure 6A,B). 

**Figure 7:**
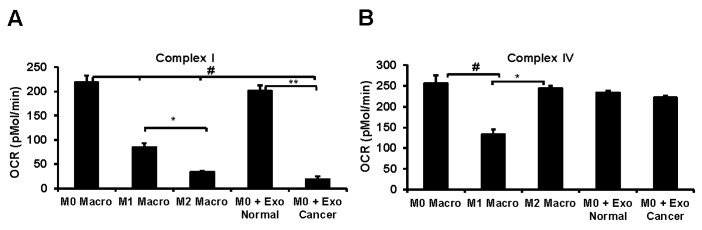
Mitochondrial respiratory chain complex activities of macrophages co-cultured with normal or tumor cell-derived exosomes. (**A**) Mitochondrial complex I activity determined in the presence of pyruvate/malate substrate after permeabilizing the plasma membrane using plasma membrane permeabilizer (PMP) in macrophages that are either polarized or co-cultured with exosomes. # *p* < 0.01 relative to M0 macrophages, * *p* <0.05 relative to M1 macrophages and ** *p* <0.001 relative to M0 macrophages treated with normal exosomes. (**B**) Mitochondrial complex IV activity determined using Ascorbate/TMPD substrate in the presence of rotenone. # *p* <0.05 relative to M0 macrophages and * *p* <0.01 relative to M1 macrophages.

M2 macrophages showed lower basal OCR, maximal OCR, and proton leak but with increased reserve capacity compared to M1 macrophages. Both M1 and M2 macrophages increased non-mitochondrial OCR compared to M0 with the highest response observed in M2 (Figure 6A,B). Interestingly, the flavoprotein/NADPH oxidase inhibitor diphenyleneiodonium chloride (DPI) inhibited the non-mitochondrial OCR suggesting an important role of these oxygen consuming mechanisms in polarized macrophages (Figure 6E). Inhibition of cellular bioenergetics was observed with macrophage co-culture of tumor cell-derived exosomes consistent with macrophage polarization to the M2 phenotype (Figure 6C,D), but exosomes derived from normal cells did not affect the bioenergetics of M0 macrophages. Interestingly, permeabilization of the plasma membrane which results in the loss of cytoplasmic factors, followed by the addition of the mitochondrial substrates pyruvate/malate, also decreased mitochondrial oxygen consumption (Figure 6F) in both M1 and M2 relative to M0 macrophages. This decrease in basal OCR was significantly higher in M2 macrophages compared to M0 and M1. Additionally, the mitochondrial respiratory complex activity decreased following co-culture with tumor cell-derived exosomes similar to M2 macrophages (Figure 7A). But complex IV activity was not altered by normal cell exosomes, tumor cell exosomes, or M2 polarization of M0 macrophages (Figure 7B). Both complex I and complex IV were suppressed during the M1 polarization of M0 macrophages (Figure 7A,B). Taken together, these data support our hypothesis that lung tumor cell-derived exosomes polarize non-committed macrophages to M2 phenotype in a syngeneic setting and also provide evidence that tumor exosomes differentiate MDSCs to M2 macrophages.

## 4. Discussion

In the present study, we demonstrate that human lung tumor cells produce EpCAM+ exosomes that express exosome specific tetraspanin markers CD9, CD63, and CD81 in addition to other protein markers TSG-101. Additionally, we show that p53 appears to be associated with exosome secretion from lung tumor cells in vitro. Our results also suggest that lung tumor cell-derived exosomes polarize non-committed M0 macrophages to an M2 phenotype independent of p53. Furthermore, we confirmed that lung tumor-derived exosomes induced M2 polarization in a syngeneic system with LLC exosomes. In in vitro studies, exosomes also polarized BMDMs and the immune sorted BM-MDSCs to M2 macrophages.

Tumor cell-derived exosomes have been reported to modulate the immune cell phenotypes in the TME [49,50]. In spite of several reports on the pro-tumorigenic role of tumor exosomes, studies evaluating their impact on immune cells have been limited. Tumor exosomes can transport a variety of bioactive molecules that promote cancer progression. It has also been shown that exosomes produced by a variety of cells carry distinctive surface proteins and content capable of signaling diverse physiological functions as reported [10,11,28,51]. Our study investigated whether lung cancer cell-derived exosomes polarize M0 macrophages into an M2 phenotype. We utilized exosomes isolated from lung tumor cells with a diameter range of 50–200 nm [22] expressing surface markers CD9, CD63, CD81, TSG-101, and EpCAM [22,52].

The p53 protein responds to a variety of stress signals and regulates a wide variety of transcription factors [42,53]. The p53 protein has been reported to regulate exosome secretion from NSCLC (H460, with a wild-type p53 allele, and H1299, with a partially deleted p53 allele) through TSAP6 (tumor suppressor-activated pathway 6) in response to stress signals [42]. Consistent with the prior studies, by using p53 null human lung cancer cell line H358, we demonstrate that p53 is associated with exosome production from human lung cancer cells, and in addition, we show that the size of lung tumor exosomes is significantly altered in the absence of p53.

A major impediment to lung cancer therapy stems from its innate resistance to chemotherapy and its ability to evade immunosurveillance. Macrophages are innate immune cells and constitute the majority of the inflammatory infiltrate in TME [8,9]. In most of the cancers, macrophages infiltrated in TME skew towards the M2 phenotype and are associated with the promotion of tumor progression and survival [8,9,54]. During the progression of NSCLC, a broad range of changes in monocytes have been reported, and monocytes further develop into TAM and promote tumor growth [54]. Numerous studies have shown that tumor-promoting factors in the TME influence the polarization of macrophages to M2 type macrophages [55,56,57]. TAMs with M2 phenotype are elevated in patients with lung cancer; however, the mechanisms underlying M2 macrophage polarization in lung cancer have not been elucidated. Here, we show that lung cancer cell-derived exosomes are internalized by M0 macrophages in a time-dependent manner. Additionally, our studies with A549 (wild-type p53 allele) and H358 (p53 null allele) exosomes suggest that lung tumor exosomes-mediated M2 polarization may not depend on the donor cell p53. In our studies, although the p53 null cell line produced less exosomes, the M2 polarization was unaffected when macrophages were co-cultured with these exosomes. Endogenous p53 activation in macrophages have been implicated in M2 macrophage polarization [58,59]. Human mutant p53 cancer cells have been shown to produce exosomes enriched with miR-1246 that promote polarization following internalization [60,61]. Therefore, irrespective of the p53 status of the exosome donor, macrophage p53 activation may influence the macrophage polarization that we see in our studies. Additionally, these exosomes could deliver miRs that promote M2 polarization in macrophages following internalization. Previously, it has been shown that melanoma exosomes result in a mixed population of the M1 and M2 phenotype [62]. In contrast, our data suggests lung cancer exosomes can polarize M2 but not M1 macrophages.

Emerging evidence suggested that BMDMs internalize tumor exosomes and show a phenotypic switch [33,34]. Ly6chi monocytes have been reported to migrate in TME and differentiate to TAM [63]. Therefore, we investigated if lung tumor exosomes polarize BM-derived monocytes to the M2 phenotype in a syngeneic system; co-cultured whole BM cells with LLC derived exosomes. Again in support of our hypothesis, lung tumor exosomes polarize BM-derived monocytes to the M2 phenotype. The clinical role for MDSCs in cancer has now emerged, and their crucial role in tumor immunology has been well documented [64,65,66]. In addition, during the progression of cancer, MDSCs have been shown to have increased suppressive activity through differentiation into TAM [47,48,66]. Consistent with this, exosomes isolated from a syngeneic cell line LLC promoted the differentiation of CD11b+Gr-1+ MDSCs to the M2 phenotype.

The phenotype switch of macrophages demonstrate the plasticity of these cells characterized by structural and functional alterations such as cellular markers, cytokine profiles, phagocytic ability and cytotoxic potential. The pattern of expression of cellular markers (CD11b, MERTK, F4/80, Arginase-1 and CD206) is consistent with the generation of M2 macrophages in response to tumor cell-derived exosomes (Figure 5). As described in other studies, we expect alterations in cytotoxic potential, phagocytic capacity or cytokine generation (IL-8, IL-10, MCP-1 and others) for tumor exosome-derived THP-1-M2 macrophages compared to the M0 phenotype [67,68]. It is not surprising that some of these responses are cell type/species-specific, for example THP-1-M2 macrophages show moderately lower phagocytic capacity but not in human monocyte-derived macrophages when compared to the respective resident macrophages [67]. It is important to note that, despite some decline, THP-1-M2 phenotype still maintains a significant level of phagocytic capacity which indeed demonstrates their ability to carry out this critical physiological function. In addition, the decline in THP-1 macrophage phagocytic response occurs only following its differentiation to the M2 phenotype, but not for the M0 phenotype, which alleviates inconsistencies in the macrophage activation response that may have occurred from the disparity in exosome uptake. In our co-culture studies, we did not observe cell death at the 10 exosome:1 cell ratio further supports the concept that tumor cell-derived exosomes induce preferential switching to THP-1-M0 macrophages to the M2 phenotype.

The cross-talk between macrophage polarization and mitochondrial metabolism has provided mechanistic insights for immuno-metabolism in the TME [69]. The pro-inflammatory M1 macrophages have suppressed mitochondrial function but are highly glycolytic to support their energy demand and to efficiently fuel the immune system through rapid turnover of the substrates subsequent to increased stability of hypoxia-inducible factor-1α (HIF-1α) and increased expression of downstream proteins [70,71,72]. Our results show that polarization of M0 macrophages to the M1 phenotype suppresses mitochondrial function and is associated with decreased activity of respiratory chain complexes I and IV (Figure 6 and Figure 7), consistent with inhibition of mitochondrial metabolism in classically activated macrophages. Polarization of M0 THP-1 macrophages to the M2 phenotype showed an overall increase in oxygen consumption; however, the mitochondrial oxygen consumption is reduced owing to lower complex I activity (Figure 6B and Figure 7A). In M2 macrophages, a significant portion of the oxygen consumption may be occurring from flavoprotein enzymes, such as nicotinamide adenine dinucleotide phosphate oxidase (NADPH oxidase), that are required for macrophage polarization and critical in the execution of phagocytic processes of macrophages [73,74]. Plasma membrane permeabilization-mediated suppression of cellular oxygen consumption in M2 macrophages in the presence of mitochondrial substrate suggests to a non-mitochondrial mechanism involving cytosolic cofactors such as NADPH. Exposure of the tumor cell-derived exosomes to macrophages in a co-culture system altered the mitochondrial and non-mitochondrial oxygen consumption similar to the M2 phenotype, consistent with the expression profiles of cellular markers determined by flow cytometry. Although the precise mechanism of this phenomenon is currently unknown, the involvement of exosomes as carriers of specific triggers such as miRNA to induce a metabolic shift to mediate macrophage polarization or signaling molecules directly to the resident macrophages have been suggested [75,76]. Evaluation of the role of the macrophage in immune response have suggested that altering the levels of mitochondrial respiratory complexes such as complex I and complex II may lead to a switch in the relative contribution of these complexes to mitochondrial respiration through a mechanism that involves phagosomal NADPH-oxidase and ROS-dependent tyrosine-kinase Fgr [77]. Inhibition of mitochondrial complex I (Figure 7A) but no change in complex IV (Figure 7B) is proposed as an adaptation in polarized macrophages to induce a shift in the proportion of NADH-FADH2 derived electrons that enter the respiratory chain secondary to the metabolic alterations of phenotype switching [78].

In summary, lung tumor cells secrete exosomes that are taken up by macrophages and differentiate into tumor-associated macrophages of the M2 phenotype, which may promote tumor growth and immune suppression. Our data demonstrate a mechanism by which lung tumor cells mediate cellular cross-talk and cause immunosuppression in TME through exosomes mediated M2 polarization and modulation of mitochondrial metabolism, thus providing beneficial insights and novel avenues for designing efficient immunotherapeutics against lung cancer.

## Figures and Tables

**Table 1:** Primers used to assess the M2 gene structure.

Gene	Primer Sequence
IL-10 Forward	AAGCCTGACCACGCTTTCTA
IL-10 Reverse	CCCAAGCCCAGAGACAAGAT
Arg1 Forward	GCCCTTTGCTGACATCCCTA
Arg1 Reverse	CGCTTGCTTTTCCCACAGAC
CHI3L1 (Ym1) Forward	AGGTCACCATTGACAGCAGC
CHI3L1 (Ym1) Reverse	ATCCTCCTGACCTCGGAACA
RETNLB (Fizz1) Forward	TCAAAAGCCAAGGCAGACCG
RETNLB (Fizz1) Reverse	AACATCCCACGAACCACAGC

## Supplementary Materials

The following are available online at https://www.mdpi.com/2073-4409/9/5/1303/s1, Figure S1. Time-dependent internalization and M2 polarization analyzed by ImageStream flow cytometry. Figure S2. Tumor cell-derived exosomes are unable to promote M1 polarization. Figure S3. Quantitation of lewis lung carcinoma (LLC) exosomes.

## Abbreviations

TME	Tumor microenvironment
MDSCs	Myeloid derived suppressor cells
LWC	Lewis lung carcinoma
NSCLC	Non-small-cell lung carcinoma
TAMs	Tumor associated macrophages
BMDMs	Bone marrow-derived macrophages
BM	Bone marrow
RPS 6	Ribosomal protein S6
OCR	Oxygen consumption rates
EpCAM	Epithelial cell adhesion molecule
TSG-101	Tumor susceptibility gene 101
PMA	Phorbol 12-myristate-13-acetate
CHI3L1	Chitinase-3-like protein 1
RETNLB	Resistin-like beta
Arg-1	Arginase-1
MerTK	Proto-oncogene tyrosine-protein kinase MER
DPI	Diphenyleneiodonium chloride
PMP	Plasma Membrane Permeabilizer
HIF-1a	Hypoxia-inducible factor-1α
NADPH oxidase	Nicotinamide adenine dinucleotide phosphate oxidase
LPS	Lipopolysaccharides
