# Supplementary material for: Lung Tumor Cell-Derived Exosomes Promote M2 Macrophage Polarization
Source: Cells. 2020 May 24;9(5):1303. doi: 10.3390/cells9051303 (PMC7290460; doi:10.3390/cells9051303)
Supplement: Supplementary file 1 [file cells-09-01303-s001.pdf]

Supplementary Figure 1

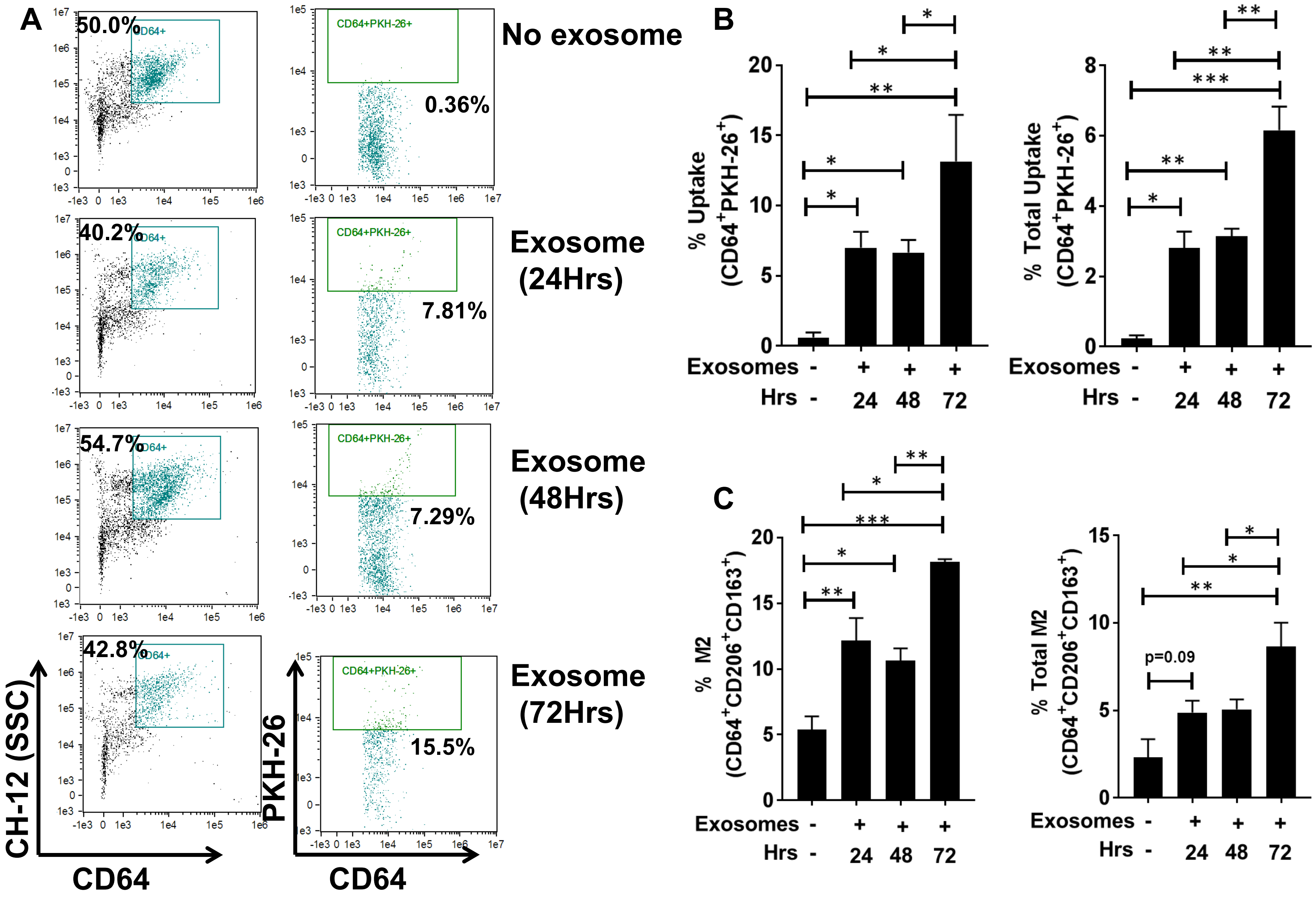

Supplementary Figure 1. Time-dependent uptake and M2 polarization analyzed by ImageStream Flow cytometry. THP-1 cells were seeded and transformed into M0 macrophages upon overnight stimulation with PMA (20-100 ng/ml). M0 macrophages were then co-cultured with PKH-26 stained A549 derived exosomes in 10:1 ratio (10 exosomes/cell) for 24 to 72 hrs. (**A**) Representative Figure for ImageStream flow cytometry analysis showing time-dependent uptake by $CD64^+$ populations analyzing by internalization of PKH-26 stained A549 derived exosomes while Cells showing no uptake and $CD64^+$ populations which indicates M0 phenotype, from the $exosome^-$ sample. (**B**) Left Panel**-** Percentage of $CD64^+PKH\text{-}26^+$ signals to show time-dependent internalization of exosomes. Right Panel**-** Total percentage of $CD64^+PKH\text{-}26^+$ signals to show time-dependent internalization of exosomes (**C**) Left Panel**-** Percentage of M2 induced by A549 cell exosomes showing time-dependent induction with the uptake. Right Panel**-** Total percentage of M2 induced by A549 cell exosomes showing time-dependent induction with the uptake.*$p < 0.05$, **$p < 0.01$***$p < 0.001$, ****$p < 0.0001$.

Supplementary Figure 2

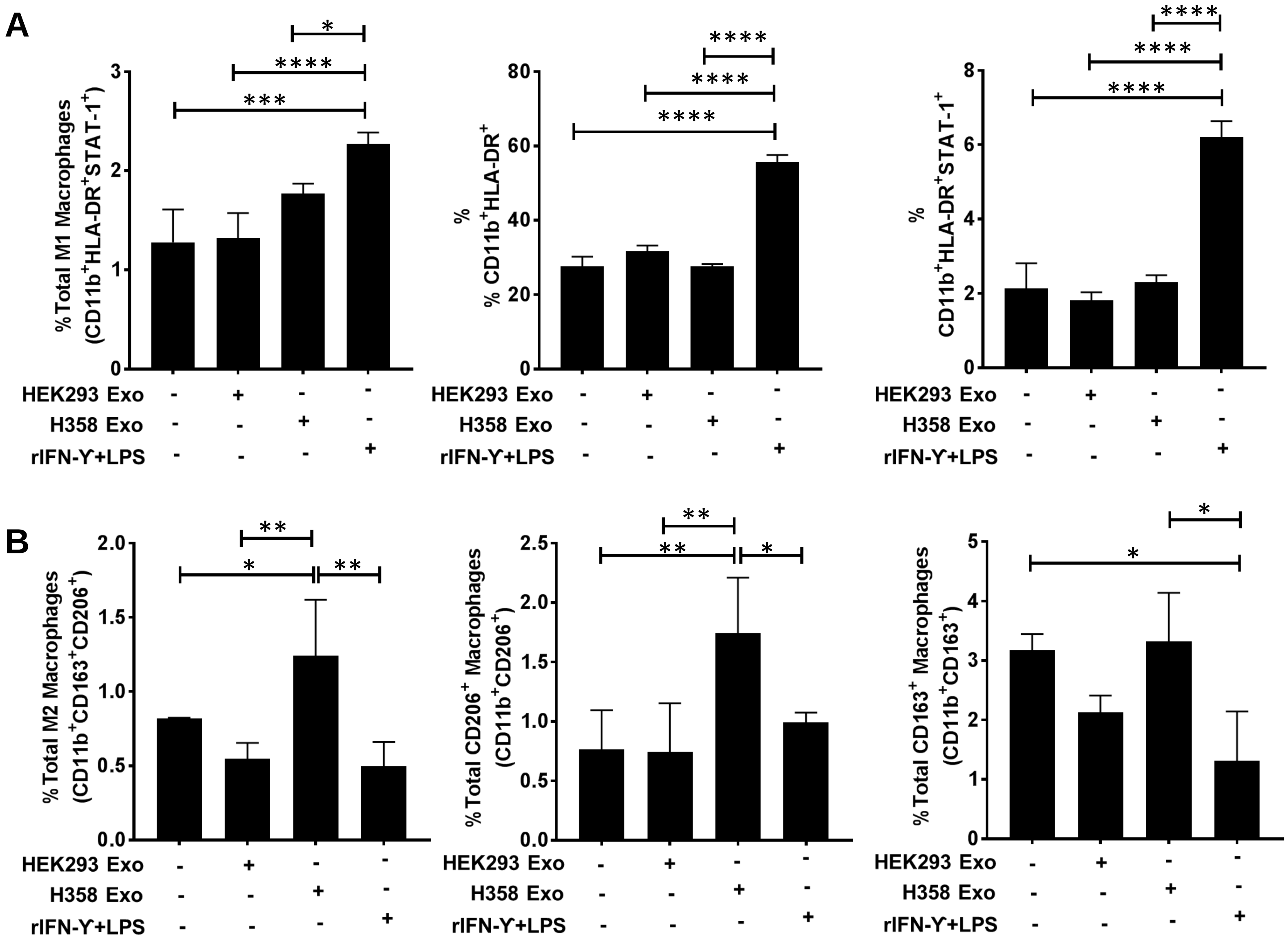

Supplementary Figure 2. Tumor cell-exosomes are unable to promote M1 polarization. THP-1 cells were seeded and transformed into M0 macrophages upon overnight stimulation with PMA (20-100 ng/ml). M0 macrophages were then co-cultured with H358, and HEK293 derived exosomes in 10:1 ratio (10 exosomes/cell) for 72 hrs. To get Positive control for M1 polarized cells, M0 macrophages were treated with rIFN-Ƴ and LPS (100 ng/ml). Cells were stained with fluorochrome-labeled CD11b, HLA-DR intracellular stain STAT-1, and CD163 and CD206. Flow cytometry was done to analyze M1 and M2 polarization. (**A**) Left to right panel- Percentage of Total M1 macrophages cells as $CD11b^{+}HLA\text{-}DR^{+}STAT\text{-}1^{+}$, parentage of $CD11b^{+}HLA\text{-}DR^{+}$, and $CD11b^{+}HLA\text{-}DR{+}STAT\text{-}1^{+}$ showing no M1 induction by tumor cells derived exosomes but it promotes (**B**) M2 polarization independent of p53.

Supplementary Figure 3

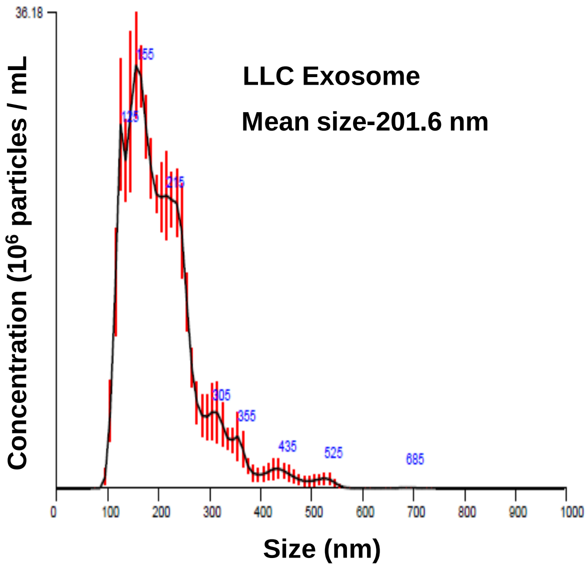

Supplementary Figure 3. Quantitation of lewis lung carcinoma (LLC) exosomes. LLC Exosomes were isolated from cultured supernatant by using differential centrifugation. Mean size and concentration of A549 derived exosome determined by Nano Sight analysis, mean size was recorded as 201.6 nm.
